# Organizational justice and illness reporting among Japanese employees with chronic diseases

**DOI:** 10.1371/journal.pone.0223595

**Published:** 2019-10-21

**Authors:** Hisashi Eguchi, Akizumi Tsutsumi, Akiomi Inoue, Yuko Kachi

**Affiliations:** Department of Public Health, Kitazato University School of Medicine, Minami-ku, Sagamihara, Kanagawa, Japan; Universtiy of Düsseldorf, GERMANY

## Abstract

**Purpose:**

This study examined the association between perceived organizational justice (i.e., procedural justice and interactional justice) and reporting of illness to one's company (illness reporting) among Japanese employees with chronic diseases.

**Methods:**

This online cross-sectional survey included 1,134 employees (aged 18–65 years) who required workplace support to combine work with disease treatment. Participants were classified into tertiles of perceived organizational justice (low, moderate, and high). Multiple logistic regression analyses were conducted to examine sex differences in the associations between perceived justice and illness reporting.

**Results:**

Males reporting perceived high levels of procedural and interactional justice had significantly higher odds of illness reporting than those with perceived low levels of procedural (odds ratio [OR] 2.62, 95% confidence interval [CI]: 1.47–4.66) and interactional justice (OR 4.34, 95% CI: 2.28–8.27). Females with perceived high levels of interactional justice had significantly higher odds of illness reporting than those with perceived low levels of interactional justice (OR 1.74, 95% CI: 1.08–2.80). There was no significant association between procedural justice and illness reporting among females.

**Conclusion:**

The findings indicate that high perceived organizational justice is positively associated with illness reporting among Japanese employees who require workplace support to combine work and disease treatment.

## Introduction

In developed countries, a substantial number of individuals of working age are diagnosed with chronic diseases [1–3]. Chronic diseases can negatively affect work participation because of disease-related limitations [4, 5]. Individuals with common chronic conditions (e.g., arthritis, diabetes, back problems, hypertension, cancer, and heart disease) are more likely to be unemployed, work fewer hours, or have reduced productivity than individuals without such conditions [6–12]. Preventing chronic conditions from worsening by coordinating work with disease prevention and treatment may lead to job retention [13, 14].

Many chronic diseases are imperceptible to others. Knowledge and understanding of illness in the workplace are needed to ensure that employees receive appropriate support from line managers and colleagues [15]. However, self-disclosure (i.e., revealing personal information about oneself) [16] is also necessary for employees to access practical and social support that will help them to effectively manage chronic conditions and perform work duties [17]. Reporting of illness to one's company (illness reporting) is the first step in promoting workplace–patient coordination during disease treatment.

Organizational justice refers to the extent to which employees are treated with fairness in the workplace [18]. The concept of justice includes a procedural component (i.e., “the extent to which decision-making procedures include input from affected parties, are consistently applied, suppress bias, and are accurate, correctable, and ethical”) and an interactional component (i.e., “polite, considerate, and fair treatment of individuals” by supervisors) [19]. Recent research suggests that organizational justice affects an individual’s decision to seek initial and ongoing care for a health issue [20–22]. A previous study showed that inferences regarding organizational justice also affected the successful implementation of processes to accommodate other employees [20]. A lack of organizational justice has been shown to be positively associated with Japanese employees refraining from seeking medical care [21], whereas organizational justice is positively associated with help-seeking behavior [22]. Organizational justice may be an important factor that influences the decisions of employees with chronic disease regarding reporting their illness to their employer.

Clarifying associations between organizational justice and illness reporting is important from a human resource management (HRM) perspective. HRM practices may impact on illness reporting because they shape the work environment and level of reciprocity between employees and their employer. HRM aims to enhance employee well-being [23]. This association is explained by two paths: a cognitive path in which high-involvement processes take “greater advantage of the skills and abilities” employees possess, and a motivational path in which involvement processes increase “workers’ satisfaction and other affective reactions” [24]. Organizational justice partially mediates the effects of HRM practices on employee well-being [25]. Illness reporting, which promotes return-to-work, may be an important part of managing an employee’s medical condition and enhancing their well-being [26]. Establishing rules to support employees with chronic diseases and providing training (including HRM) to improve awareness of these rules may encourage employees with chronic diseases to take action to access needed support [27].

This study aimed to examine the association between organizational justice (i.e., procedural and interactional justice) and illness reporting among Japanese employees with chronic diseases. We hypothesized that individuals who perceived higher levels of organizational justice would be more likely to report their illness to their employer. There is evidence of sex-based differences in occupational and working time distribution in Japan [28]; therefore, separate analyses were conducted for male and female employees.

## Materials and methods

### Participants and survey method

A cross-sectional, online survey was conducted in February 2018 with participants registered with a Japanese web survey company, Macromill, Inc [29]. The web survey company regularly collects registrants’ information about chronic diseases. The diseases included: acquired immune deficiency syndrome, Alzheimer’s-type dementia, aplastic anemia, bipolar disorder, cancer, cerebral hemorrhage, cerebral infarction, cerebrovascular dementia, chronic renal failure, Crohn’s disease, depression, fibromyalgia, hemophilia, Lewy body dementia, myasthenia gravis, myelodysplastic syndrome, myelofibrosis, metabolic endocrine disease, mixed connective tissue disease, multiple sclerosis, Parkinson’s disease, rheumatoid arthritis, schizophrenia, subarachnoid hemorrhage, systemic lupus erythematosus, and ulcerative colitis.

In total, 89,874 people aged 18–65 years with chronic diseases were randomly invited to participate in a screening survey for the present study. Participants who answered “yes” to three screening questions were invited to complete the survey: 1) “Are you currently suffering from any diseases or disorders that are not curable over a short period and require repetitive/continuous treatment (e.g., cancer, stroke, cardiac disease, diabetes, hepatitis, connective tissue disease, intractable neurological disease)?”; 2) “Are you currently working?”; and 3) “Do you currently need some support from the company you work for in order to continue your job while undergoing appropriate medical treatment?” A small financial incentive was offered for responding to the survey (equivalent to a few US dollars). The web survey company then invited randomly selected registrants to complete the survey. For financial reasons, recruitment ceased when the number of participants reached a set target. The sex ratio was 1:1.

### Organizational justice

Organizational justice was measured using the Japanese version of the Organizational Justice Questionnaire (OJQ) [18, 19, 30]. The OJQ comprises a seven-item procedural justice subscale and a six-item interactional justice subscale. Both subscales are measured on a five-point Likert-type scale from 1 = “strongly disagree” to 5 = “strongly agree.” The total score for each OJQ subscale was calculated by averaging the item scores (score range 1–5). In this sample, the Cronbach’s alpha coefficients were 0.93 for the procedural justice subscale and 0.96 for the interactional justice subscale among male employees, and 0.92 for the procedural justice subscale and 0.95 for the interactional justice subscale among female employees.

### Illness reporting

Participants’ illness reporting to their employer was determined by responses to the question “Did you report your illness to the company (e.g., your manager, personnel department, occupational physician)?” Possible responses were “yes” or “no.”

### Potential confounders

Demographic and occupational characteristics were considered potential confounders and measured using a self-administered questionnaire. Demographic characteristics were sex, age, residential area, marital status, having children, household income, and educational attainment. Age was classified into five groups: 18–29, 30–39, 40–49, 50–59, and 60–65 years. Residential area was classified into eight groups based on administrative divisions: Hokkaido (Sapporo), Tohoku (Sendai), Kanto (Tokyo), Chubu (Nagoya), Kinki (Osaka), Chugoku (Hiroshima), Shikoku (Matsuyama), and Kyushu/Okinawa (Fukuoka). Marital status was classified as unmarried or married, and having children was classified as “yes” or “no.” Household income was classified into three groups: low (<3 million yen/year), middle (3–5 million yen/year), and high (>5 million yen/year) (1 US dollar = approximately 110 Japanese yen at the time). Educational attainment was classified into three categories: junior high school or high school, technical college or junior college, and university or graduate school.

Occupational characteristics comprised weekly working hours, employment status, occupation, employment as a registered disabled person, company size, and type of industry. Weekly working hours were categorized as ≤40, 41–60, and ≥61 hours. Employment status was assessed using the six options from the Japanese labor force statistics [31]: manager/executive, regular employee (full-time employee), contract employee (part-time employee), part-time laborer, dispatched employee, and temporary/day laborer. We dichotomized responses as regular employment (manager/executive and regular employee) and non-regular employment (contract employee/part-time employee, part-time laborer, dispatched employee, and temporary/day laborer). We classified occupation based on skill level and skill specialization using the International Standard Classification of Occupations (ISCO) [32]. Participants were asked whether they were currently employed as a manager; those who were not managers were asked whether they were professional, technical, clerical, service, or manual workers. We further classified the ISCO groups according to participants’ employment characteristics, including levels of authority, specialized knowledge/expertise, and career opportunities in each occupation group. Based on these occupational groups, participants were divided into three occupational categories: 1) manager; 2) white collar (professional/technical/clerical/service); and 3) blue collar (manual). Employment as a registered disabled person was assessed with the question: “Were you hired as a registered disabled person?” Response options were “yes” or “no.” Company size was classified into six groups (<10, 10–49, 50–299, 300–999, and ≥1000 employees, and public sector). Type of industry was dichotomized as manufacturing or non-manufacturing (including commerce, finance, and social welfare).

### Ethical approval

The study aims and protocol were approved in 2018 by the Kitasato University Medical Ethics Organization (B17-160). All study procedures were consistent with the ethical standards of the responsible committees on human experimentation (institutional and national) and with the Helsinki Declaration of 1975, as revised in 2000 [5]. Informed consent to participate in this study was obtained from all participants. Participants were informed in advance that their participation was strictly voluntary and that all information provided would remain confidential. Those who consented to participate were able to access a designated website on verification of their personal information, after which they could complete the survey online. Participants had the option of not responding to any part of the questionnaire, and could discontinue participation at any point.

### Statistical analysis

Logistic regression was used to examine associations between organizational justice (i.e., procedural or interactional justice) and illness reporting. Analyses were performed stratified by sex and with the sexes combined. In the regression analyses, we first conducted crude analyses of the association between organizational justice and illness reporting. Next, we adjusted for age, residential area, marital status, having children, household income, educational attainment, weekly working hours, employment status, occupation, employment as a registered disabled person, company size, and industry. All analyses were performed using Stata 15SE (StataCorp, College Station, TX, USA), with statistical significance set at p < 0.05.

## Results

In total, 1,134 individuals (567 males and 567 females) participated in this study. For males, the average (standard deviation) score for perceived procedural justice was 3.03 (0.92) and that for perceived interactional justice was 3.08 (1.00). For females, the average (standard deviation) score for perceived procedural justice was 2.95 (0.86) and that for perceived interactional justice was 3.13 (1.01). Participants’ background characteristics are shown in Table 1. Male participants who had reported their illness to their employer were significantly older and had higher perceived procedural and interactional justice than those who did not report their illness. Female participants who reported their illness to their employer were more likely to be regular or disabled employees and had higher perceived interactional justice than females who did not report their illness. The percentages of male participants’ that reported their illness to their employer were 16.9% and 23.6% higher than those that did not report their illness in the upper tertiles of procedural and interactional justice, respectively. The corresponding figures among female participants in the upper tertiles of procedural and interactional justice were 3.4% and 12.6%, respectively.

**Table 1:** Detailed characteristics of participants (n = 1,134).

			Total (n = 1,134)																				
			Reported their illness to their employer																				
			Yes (n = 454)					No (n = 113)				P value^a^											
			n	(	%	)		n	(	%	)												
Age																							
	18–29		59	(	6.8	)		17	(	6.4	)	0.724											
	30–39		164	(	18.9	)		55	(	20.6	)												
	40–49		268	(	30.9	)		91	(	34.1	)												
	50–59		294	(	33.9	)		83	(	31.1	)												
	60–65		82	(	9.5	)		21	(	7.9	)												
Residential area																							
	Hokkaido (Sapporo)		45	(	5.2	)		14	(	5.2	)	0.458											
	Tohoku (Sendai)		56	(	6.5	)		17	(	6.4	)												
	Kanto (Tokyo)		299	(	34.5	)		97	(	36.3	)												
	Chubu (Nagoya)		160	(	18.5	)		41	(	15.4	)												
	Kinki (Osaka)		158	(	18.2	)		60	(	22.5	)												
	Chugoku (Hiroshima)		41	(	4.7	)		11	(	4.1	)												
	Shikoku (Matsuyama)		33	(	3.8	)		4	(	1.5	)												
	Kyushu/Okinawa (Fukuoka)		75	(	8.7	)		23	(	8.6	)												
Marital status																							
	Married		466	(	53.8	)		127	(	47.6	)	0.077											
	Unmarried		401	(	46.3	)		140	(	52.4	)												
Having children																							
	Yes		410	(	47.3	)		122	(	45.7	)	0.648											
	No		457	(	52.7	)		145	(	54.3	)												
Household income (million yen per year)																							
	Low (<3 million yen/year)		127	(	14.7	)		63	(	23.6	)	<0.001											
	Middle (3-5 million yen/year)		226	(	26.1	)		89	(	33.3	)												
	High (>5 million yen/year)		514	(	59.3	)		115	(	43.1	)												
Educational attainment																							
	Junior high school or high school		174	(	20.1	)		72	(	27.0	)	0.020											
	Technical college or junior college		193	(	22.3	)		65	(	24.3	)												
	University and graduate school		500	(	57.7	)		130	(	48.7	)												
Weekly working hour																							
	≤40		567	(	65.4	)		166	(	62.2	)	0.622											
	41–60		263	(	30.3	)		88	(	33.0	)												
	≥61		37	(	4.3	)		13	(	4.9	)												
Employment status																							
	Regular		727	(	83.9	)		196	(	73.4	)	<0.001											
	Non-regular		140	(	16.2	)		71	(	26.6	)												
Occupation																							
	Manager		42	(	4.8	)		11	(	4.12	)	0.429											
	White collar		732	(	84.4	)		220	(	82.4	)												
	Blue collar		93	(	10.7	)		36	(	13.5	)												
Employment as a disabled person																							
	Yes		121	(	14.0	)		20	(	7.5	)	0.005											
	No		746	(	86.0	)		247	(	92.5	)												
Company size																							
	<10		98	(	11.3	)		23	(	8.6	)	0.082											
	10–49		106	(	12.2	)		47	(	17.6	)												
	50–299		201	(	23.2	)		73	(	27.3	)												
	300–999		138	(	15.9	)		40	(	15.0	)												
	1000<		259	(	29.9	)		70	(	26.2	)												
	Public service		65	(	7.5	)		14	(	5.2	)												
Industry																							
	Manufacturing		710	(	81.9	)		223	(	83.5	)	0.542											
	Non-manufacturing		157	(	18.1	)		44	(	16.5	)												
Procedural justice (1-5)																							
	Low (1.00—2.57)		237	(	27.3	)		96	(	36.0	)	0.007											
	Middle (2.71-3.14)		289	(	33.3	)		91	(	34.1	)												
	High (3.29-5.00)		341	(	39.3	)		80	(	30.0	)												
Interactional justice (1-5)																							
	Low (1.00—2.57)		236	(	27.2	)		106	(	39.7	)	<0.001											
	Middle (2.71-3.14)		288	(	33.2	)		101	(	37.8	)												
	High (3.29-5.00)		343	(	39.6	)		60	(	22.5	)												
			Men (n=567)												Women (n=567)								
			Reported their illness to their employer																				
			Yes (n = 454)					No (n = 113)				P value^a^		Yes (n = 413)					No (n = 154)				P value^a^
			n	(	%	)		n	(	%	)			n	(	%	)		n	(	%	)	
Age																							
	18–29		2	(	0.4	)		5	(	4.4	)	0.007		57	(	13.8	)		12	(	7.8	)	0.075
	30–39		40	(	8.8	)		8	(	7.1	)			124	(	30.0	)		47	(	30.5	)	
	40–49		140	(	30.8	)		27	(	23.9	)			128	(	31.0	)		64	(	41.6	)	
	50–59		197	(	43.4	)		55	(	48.7	)			97	(	23.5	)		28	(	18.2	)	
	60–65		75	(	16.5	)		18	(	15.9	)			7	(	1.7	)		3	(	2.0	)	
Residential area																							
	Hokkaido (Sapporo)		23	(	5.1	)		8	(	7.1	)	0.664		22	(	5.3	)		6	(	3.9	)	0.765
	Tohoku (Sendai)		27	(	6.0	)		8	(	7.1	)			29	(	7.0	)		9	(	5.8	)	
	Kanto (Tokyo)		165	(	36.3	)		42	(	32.2	)			134	(	32.5	)		55	(	35.7	)	
	Chubu (Nagoya)		91	(	20.0	)		18	(	15.9	)			69	(	16.7	)		23	(	14.9	)	
	Kinki (Osaka)		79	(	17.4	)		23	(	20.4	)			79	(	19.1	)		37	(	24.0	)	
	Chugoku (Hiroshima)		14	(	3.1	)		4	(	3.5	)			27	(	6.5	)		7	(	4.6	)	
	Shikoku (Matsuyama)		19	(	4.2	)		1	(	0.9	)			14	(	3.4	)		3	(	2.0	)	
	Kyushu/Okinawa (Fukuoka)		36	(	7.9	)		9	(	8.0	)			39	(	9.4	)		14	(	9.1	)	
Marital status																							
	Married		302	(	66.5	)		65	(	57.5	)	0.073		164	(	39.7	)		62	(	40.3	)	0.905
	Unmarried		152	(	33.5	)		48	(	42.5	)			249	(	60.3	)		92	(	59.7	)	
Having children																							
	Yes		256	(	56.4	)		59	(	52.2	)	0.424		154	(	37.3	)		63	(	40.9	)	0.430
	No		198	(	43.6	)		54	(	47.8	)			259	(	62.7	)		91	(	59.1	)	
Household income (million yen per year)																							
	Low (<3 million yen/year)		46	(	10.1	)		22	(	19.5	)	<0.001		81	(	19.6	)		41	(	26.6	)	0.103
	Middle (3-5 million yen/year)		111	(	24.5	)		43	(	38.1	)			115	(	27.9	)		46	(	29.9	)	
	High (>5 million yen/year)		297	(	65.4	)		48	(	42.5	)			217	(	52.5	)		67	(	43.5	)	
Educational attainment																							
	Junior high school or high school		91	(	20.0	)		39	(	34.5	)	0.004		83	(	20.1	)		33	(	21.4	)	0.901
	Technical college or junior college		59	(	13.0	)		14	(	12.4	)			134	(	32.5	)		51	(	33.1	)	
	University and graduate school		304	(	67.0	)		60	(	53.1	)			196	(	47.5	)		70	(	45.5	)	
Weekly working hour																							
	≤40		284	(	62.6	)		61	(	54.0	)	0.246		283	(	68.5	)		105	(	68.2	)	0.907
	41–60		146	(	32.2	)		45	(	39.8	)			117	(	28.3	)		43	(	27.9	)	
	≥61		24	(	5.3	)		7	(	6.2	)			13	(	3.2	)		6	(	3.9	)	
Employment status																							
	Regular		397	(	87.4	)		85	(	75.2	)	0.001		330	(	79.9	)		111	(	72.1	)	0.046
	Non-regular		57	(	12.6	)		28	(	24.8	)			83	(	20.1	)		43	(	27.9	)	
Occupation																							
	Manager		33	(	7.3	)		8	(	7.1	)	0.225		9	(	2.2	)		3	(	2.0	)	0.984
	White collar		378	(	83.3	)		88	(	77.9	)			354	(	85.7	)		132	(	85.7	)	
	Blue collar		43	(	9.5	)		17	(	15.0	)			50	(	12.1	)		19	(	12.3	)	
Employment as a disabled person																							
	Yes		53	(	11.7	)		9	(	8.0	)	0.258		68	(	16.5	)		11	(	7.1	)	0.004
	No		401	(	88.3	)		104	(	92.0	)			345	(	83.5	)		143	(	92.9	)	
Company size																							
	<10		45	(	9.9	)		13	(	11.5	)	0.564		53	(	12.8	)		10	(	6.5	)	0.053
	10–49		55	(	12.1	)		16	(	14.2	)			51	(	12.4	)		31	(	20.1	)	
	50–299		104	(	22.9	)		30	(	26.6	)			97	(	23.5	)		43	(	27.9	)	
	300–999		69	(	15.2	)		20	(	17.7	)			69	(	16.7	)		20	(	13.0	)	
	1000<		142	(	31.3	)		28	(	24.8	)			117	(	28.3	)		42	(	27.3	)	
	Public service		39	(	8.6	)		6	(	5.3	)			26	(	6.3	)		8	(	5.2	)	
Industry																							
	Manufacturing		345	(	76.0	)		91	(	80.5	)	0.306		365	(	88.4	)		132	(	85.7	)	0.391
	Non-manufacturing		109	(	24.0	)		22	(	19.5	)			48	(	11.6	)		22	(	14.3	)	
Procedural justice (1-5)																							
	Low (1.00—2.57)		111	(	24.5	)		42	(	37.2	)	0.002		126	(	30.5	)		54	(	35.1	)	0.569
	Middle (2.71-3.14)		158	(	34.8	)		44	(	38.9	)			131	(	31.7	)		47	(	30.5	)	
	High (3.29-5.00)		185	(	40.8	)		27	(	23.9	)			156	(	37.8	)		53	(	34.4	)	
Interactional justice (1-5)																							
	Low (1.00—2.57)		115	(	25.3	)		48	(	42.5	)	<0.001		121	(	29.3	)		58	(	37.7	)	0.020
	Middle (2.71-3.14)		166	(	36.6	)		49	(	43.4	)			122	(	29.5	)		52	(	33.8	)	
	High (3.29-5.00)		173	(	38.1	)		16	(	14.5	)			170	(	41.2	)		44	(	28.6	)	

^a^ Fisher’s exact test was conducted.

Table 2 shows the results of the multiple logistic regression analyses for the associations between organizational justice and illness reporting. We observed significantly higher odds of illness reporting among participants that reported high levels of perceived procedural (adjusted odds ratio [OR] 1.60, 95% confidence interval [CI]: 1.12–2.29, p = 0.010) and interactional justice (adjusted OR 2.41, 95% CI: 1.67–3.49, p < 0.001) than among those who reported low levels of perceived procedural and interactional justice.

**Table 2:** Multiple logistic regression analysis for associations between perceived organizational justice and illness reporting among Japanese employees with chronic diseases (n = 1,134).

		Crude OR	95% CI	Adjusted OR^a^	95% CI
Procedural justice					
	Low	ref		ref	
	Middle	1.29	0.92–1.80	1.22	0.87–1.73
	High	1.73	1.23–2.42	1.60	1.12–2.29
Interactional justice					
	Low	ref			
	Middle	1.28	0.93–1.77	1.19	0.85–1.67
	High	2.57	1.80–3.67	2.41	1.67–3.49

OR, odds ratio; CI, confidence interval.

a Adjusted OR: adjusted for age, sex, residential area, marital status, having children, household income, educational attainment, employment as a disabled person, weekly working hour, employment status, occupation, company size, and industry.

Table 3 shows the results of the multiple logistic regression analyses for the associations between organizational justice and illness reporting by sex. Males reporting high levels of perceived procedural and interactional justice had significantly higher odds of reporting their illness to their employer than those with low levels of perceived procedural and interactional justice (OR 2.62, 95% CI: 1.47–4.66, p < 0.001 and OR 4.34, 95% CI: 2.28–8.27, p = 0.019, respectively). Female participants reporting high levels of perceived interactional justice had significantly higher odds of reporting their illness to their employer than those with low perceived interactional justice (OR 1.74, 95% CI: 1.08–2.80). However, the association between procedural justice and illness reporting was not significant for female employees (OR 1.11, 95% CI: 0.69–1.79).

**Table 3:** Multiple logistic regression analysis for associations between perceived organizational justice and illness reporting among Japanese employees with chronic diseases by sex (n = 1,134).

		Crude OR	95% CI	Adjusted OR^a^	95% CI
Males (n = 567)					
Procedural justice					
	Low	ref		ref	
	Middle	1.36	0.83–2.21	1.35	0.80–2.28
	High	2.59	1.51–4.44	2.62	1.47–4.66
Interactional justice					
	Low	ref		ref	
	Middle	1.41	0.89–2.25	1.35	0.82–2.21
	High	4.51	2.44–8.33	4.34	2.28–8.27
					
Females (n = 567)					
Procedural justice					
	Low	ref		ref	
	Middle	1.19	0.75–1.89	1.06	0.66–1.71
	High	1.26	0.81–1.97	1.11	0.69–1.79
Interactional justice					
	Low	ref		ref	
	Middle	1.12	0.71–1.77	1.03	0.64–1.65
	High	1.85	1.17–2.92	1.74	1.08–2.80

OR, odds ratio; CI, confidence interval.

^a^ Adjusted OR: adjusted for age, residential area, marital status, having children, household income, educational attainment, employment as a disabled person, weekly working hours, employment status, occupation, company size, and industry.

## Discussion

This study investigated the associations between organizational justice and illness reporting among employees with chronic diseases across Japan. Most participants (76.5%) had informed their employer about their chronic illness. Male participants that had high perceived procedural and interactional justice had significantly higher odds of reporting their illness than those with low perceived procedural and interactional justice. Among female participants, those with high perceived interactional justice had significantly higher odds of reporting their illness than those with low perceived interactional justice, but the association between procedural justice and illness reporting was not significant. This result may contribute to clarifying the effect of HRM on employees’ well-being.

Interactional justice, which pertains to the fairness of interpersonal treatment by supervisors [33], was significantly associated with illness reporting. This suggests that employees’ interactions with supervisors affect proactive behavior toward workplace–patient coordination and disease treatment among employees with chronic diseases. This result was consistent with previous evidence that showed organizational justice was positively associated with employees’ help-seeking behavior [21, 22]. Trust and mutual respect are important components of interactional justice as expressed in interactions with supervisors, and play a central role in this association [34]. Interactional unfairness is a clear and direct signal of rejection and devaluation [35, 36]. Improvements in workplace interactional justice may generate a climate that facilitates illness reporting among employees with chronic diseases.

Procedural justice relates to the fairness of the decision-making process and involves consideration of the interests of those affected by decisions. Our study showed high perceived procedural justice was significantly associated with illness reporting among males (as expected), but not among females. In Japan, job performance is traditionally evaluated in terms of a high level of commitment to the employer, which is demonstrated by acceptance of long working hours, frequent relocation, and length of service [37, 38]. A possible consequence of this type of work culture is the devaluation of employees whose time commitment may be limited by treatments and side effects. Moreover, in Japan, 59.4% of male employees are interested in promotion, compared with 10.4% of female employees [39]. Therefore, male employees may be more sensitive to fairness in the workplace than female employees.

It is unknown whether the levels of organizational justice in the studied population were higher or lower than those in the general working population. Previous Japanese studies that investigated employees from several companies reported average procedural justice scores of 3.00 and 3.16 for males and 2.93 and 3.28 for females, and interactional justice scores of 3.27 and 3.56 for males and 3.14 and 3.41 for females [30, 40]. The levels of organizational justice in the present study were similar to one of these studies [30] but lower than the other [40]. Different levels of perceived organizational justice may be expected between workers with and without chronic diseases, because the presence of chronic diseases may impact employees’ perceptions of workplace psychosocial factors [41, 42]. The observed inconsistencies in the results may be explained by the fact that previous Japanese studies did not exclude workers with chronic diseases from the respective datasets.

This study had some limitations. First, our study population required Internet access to complete the survey and therefore might have been more aware of the balance between work and treatment through access to online information [43, 44]. Our results are not completely generalizable to workers without Internet access, or to other countries and settings. Second, the possibility of survival bias should be considered in this study because of its cross-sectional design. Employees with low perceived organizational justice who did not report their illness may be more likely to leave their job than those with high perceived organizational justice who reported their illness. Third, further studies are needed to evaluate whether other confounding factors may explain the observed attenuation in the association between organizational justice and illness reporting. For example, we did not collect information about individual diseases or their severity, or participants’ work ability. Fourth, this study was cross-sectional and causal associations cannot be assumed. Further interventional research is needed to clarify potential causal associations between organizational justice and illness reporting among Japanese employees with chronic diseases. Finally, we could not confirm the exact diagnosis of participants who judged that their work capacity was limited because of symptoms related to a chronic disease or side effects of their treatment.

In conclusion, the present study provided evidence that high levels of perceived organizational justice are positively associated with illness reporting in Japanese employees independently of demographic and socioeconomic characteristics. Employees with chronic illness may need workplace support to effectively combine work and disease treatment.

## Supporting information

S1 File Questionnaire. (DOCX)
