# Supplementary material for: Organizational justice and illness reporting among Japanese employees with chronic diseases
Source: PLoS One. 2019 Oct 21;14(10):e0223595. doi: 10.1371/journal.pone.0223595 (PMC6802873; doi:10.1371/journal.pone.0223595)
Supplement: S1 File — (DOCX) [file pone.0223595.s001.docx]

**Questionnaire**

**Screening items**

1. Are you currently suffering from diseases or disorders that are not cured over a short period and require repetitive/continuous treatment (e.g. cancer, stroke, cardiac disease, diabetes, hepatitis, connective tissue disease, intractable neurological disease)?

□Yes □No

2. Are you currently working?

□Yes □No

3. Currently, do you need some support from the company you work for in order to continue your job while undergoing appropriate medical treatment?

□Yes □No

**Illness reporting**

Did you report to the company (your boss, personnel department, occupational physician, manager, etc.) about your illness?

□Yes, I reported. □No, I didn’t report.

**Organizational justice**

1) These next statements are about decision making at your workplace. Please read each statement carefully, and please tick the most appropriate response.

|  | Strongly disagree. | Disagree. | Neutral | Agree. | Strongly agree. |
| --- | --- | --- | --- | --- | --- |
| 1. Decisions are made based on accurate information. | 1 | 2 | 3 | 4 | 5 |
| 2. People are provided opportunities to appeal or challenge decisions they find unsuccessful. | 1 | 2 | 3 | 4 | 5 |
| 3. All sides affected by the decision are represented in decision making. | 1 | 2 | 3 | 4 | 5 |
| 4. Decisions are made with consistency (the rules are the same for every employee). | 1 | 2 | 3 | 4 | 5 |
| 5. The concerns of all those affected by the decision are heard before decision making. | 1 | 2 | 3 | 4 | 5 |
| 6. Feedback is collected regarding the decision and its implementation. | 1 | 2 | 3 | 4 | 5 |
| 7. It is possible to requests for clarification or additional information about the decision. | 1 | 2 | 3 | 4 | 5 |

2) These next statements are about your boss’ attitude and behavior. Please read each statement carefully, and please tick the most appropriate response.

|  | Strongly disagree. | Disagree. | Neutral | Agree. | Strongly agree. |
| --- | --- | --- | --- | --- | --- |
| 1. Our supervisor considers our viewpoint. | 1 | 2 | 3 | 4 | 5 |
| 2. Our supervisor is able to suppress personal biases. | 1 | 2 | 3 | 4 | 5 |
| 3. Our supervisor provides us with timely feedback about the decisions and their implications. | 1 | 2 | 3 | 4 | 5 |
| 4. Our supervisor treats us with kindness and consideration. | 1 | 2 | 3 | 4 | 5 |
| 5. Our supervisor shows concern for our rights as an employee. | 1 | 2 | 3 | 4 | 5 |
| 6. Our supervisor takes steps to deal with us in a truthful manner. | 1 | 2 | 3 | 4 | 5 |

**Sociodemographic item**

| 1. How old are you? | _____ years | | |
| --- | --- | --- | --- |
| 2. Are you male or female? | 1. Male  2. Female | | |
| 3. What is your marital status? | 1. Yes  2. No | | |
| 4. Which of these was the last school you attended? | 1. Junior high school  2. Senior high school  3. Vocational college  4. Junior college, higher technical college  5. University  6. Graduate school | | |
| 5. Which of the following best describes your current work?  1. Managerial work (please tick if you are ranked as a section chief or higher)  2. Specialist work (researcher, engineer, computer engineer, doctor, nurse, teacher, etc.)  3. Technical work (electrician, computer technician, nutritionist, etc.)  4. Administrative work (general administration, accounting, secretary, keypuncher, etc.)  5. Services work (sales person, security, waitress, childcare worker, carer, etc.)  6. Production work requiring certain skills (construction, machinery repair, maintenance, handicrafts, etc.)  7. Production work operating machinery (machine operator, car driver, etc.)  8. Production work involving mostly physical operations (packing, shipping, cleaning, etc.)  9. Other work | | | |
| 6. On average, how many hours do you work each week (including overtime)? | | | |
| 7. What is your approximate household income (including tax)? | | | 1. Less than 2.99 million yen  2. 3.00-4.99 million yen  3. 5.00-7.99 million yen  4. 8.00-9.99 million yen  5. 10.00-14.99 million yen  6. 15.00 million yen or more |
| 8. Do you have children? | | | 1. Yes  2. No |
| 9. Where is your residential area? | | | 1. Hokkaido  2. Tohoku  3. Kanto  4. Chubu  5. Kinki  6. Chugoku  7. Shikoku  8. Kyushu/Okinawa |
| 10. What is your employment status? | | | 1. Manager/executive  2. Regular employee (full-time employee)  3. Contract employee (part-time employee)  4. Part-time labourer  5. Dispatched employee  6. Temporary/day laborer  7. Others |
| 11. Are you employed as a disabled person? | | 1. Yes  2. No | |
| 12. What is the approximate total number of employees your company has at all the locations in Japan? | | 1. 10-29  2. 30-49  3. 50-99  4. 100-299  5. 300-999  6. 1,000-4,999  7. 5,000 or more  8. Public sector | |
| 13. Which of the following categories best describes the industry you primarily work in. | | 1. Agriculture  2. Fisheries  3. Mining  4. Construction  5. Manufacturing process  6. Electricity and gas  7. Information  8. Transport  9. Wholesale and retail  10. Finance  11. Real estate and rental  12. Research and professional services  13. Accommodations and dining services  14. Amusement services  15. Education  16. Medical and welfare  17. Compound services  18. Other services  19. Government  20. Others | |

**質問票**

**スクリーニング項目**

1．あなたは、今、がん、脳卒中、心臓病、糖尿病、肝炎、膠原病、神経難病　　　　　など、反復・継続して治療が必要で、短期で治癒しない疾病や障害を抱えて　　いますか。

□はい　□いいえ

2．あなたは、今、仕事をしていますか。

□はい　□いいえ

3．あなたは、今、適切な治療を受けながら、仕事を継続するにあたって、　　　　会社から何かしらの支援が必要ですか。

□はい　□いいえ

**持病の報告**

あなたは、ご自身の病気ことを会社（上司や人事、産業医、経営者など）に報告していますか。

□報告している　□報告していない

**組織的公正**

**1) あなたの職場の意思決定について，最もあてはまるもの1つにチェックをつけてください。**

|  | 全く当てはまらない | あまり当てはまらない | どちらともいえない | やや当てはまる | 非常に当てはまる |
| --- | --- | --- | --- | --- | --- |
|  |  |  |  |  |  |
| 1. 意思決定は正確な情報に基づいてなされている。 | ❑_1_ | ❑_2_ | ❑_3_ | ❑_4_ | ❑_5_ |
| 1. 決めたことがうまくいかなかった場合に意見を述べたり，異議を申し立てたりする機会が与えられている。 | ❑_1_ | ❑_2_ | ❑_3_ | ❑_4_ | ❑_5_ |
| 1. 意思決定によって影響を受ける全ての関係者が，意思決定に参加している。 | ❑_1_ | ❑_2_ | ❑_3_ | ❑_4_ | ❑_5_ |
| 1. 意思決定は一貫している(全ての従業員に対し規則が同様に適用される)。 | ❑_1_ | ❑_2_ | ❑_3_ | ❑_4_ | ❑_5_ |
| 1. 意思決定によって影響を受ける全ての人たちの考えが，意思決定をする前に聞かれている。 | ❑_1_ | ❑_2_ | ❑_3_ | ❑_4_ | ❑_5_ |
| 1. 意思決定やそれに基づく実施の結果について，事後に意見や情報が集められている。 | ❑_1_ | ❑_2_ | ❑_3_ | ❑_4_ | ❑_5_ |
| 1. 意思決定について分からないことがあれば，説明や追加情報を要求することが可能である。 | ❑_1_ | ❑_2_ | ❑_3_ | ❑_4_ | ❑_5_ |

**2) あなたの上司の態度や行動について，最もあてはまるもの1つにチェックをつけてください。**

|  | 全く当てはまらない | あまり当てはまらない | どちらともいえない | やや当てはまる | 非常に当てはまる |
| --- | --- | --- | --- | --- | --- |
|  |  |  |  |  |  |
| 1. 上司は私たちの考え方を考慮してくれる。 | ❑_1_ | ❑_2_ | ❑_3_ | ❑_4_ | ❑_5_ |
| 1. 上司は独りよがりなものの見方をしないようにすることができる。 | ❑_1_ | ❑_2_ | ❑_3_ | ❑_4_ | ❑_5_ |
| 1. 上司は意思決定やその影響について，タイミングよく情報を提供してくれる。 | ❑_1_ | ❑_2_ | ❑_3_ | ❑_4_ | ❑_5_ |
| 1. 上司は親切心と思いやりをもって私たちに接してくれる。 | ❑_1_ | ❑_2_ | ❑_3_ | ❑_4_ | ❑_5_ |
| 1. 上司は私たちの従業員としての権利に対して理解を示してくれる。 | ❑_1_ | ❑_2_ | ❑_3_ | ❑_4_ | ❑_5_ |
| 1. 上司は誠実な態度で，私たちに対応してくれる。 | ❑_1_ | ❑_2_ | ❑_3_ | ❑_4_ | ❑_5_ |

**あなた自身のことについてお教えください。**

| 1. あなたの年齢をお答えください。 | （　　　　　）歳 | | |
| --- | --- | --- | --- |
| 2. あなたの性別をお答えください。 | 1. 男性  2. 女性 | | |
| 3. あなたは結婚していますか。 | 1. はい  2. いいえ | | |
| 4. 最終の学校教育歴をお答えください。 | 1．中学校卒業  2．高校中退・卒業  3．短大・高専・専門学校中退・卒業  4．大学中退・卒業  5．大学院中退・修了 | | |
| 5. あなたの現在の仕事は以下のどれに一番近いですか。  　1.　管理職 (課長職以上の方はここをチェックしてください)  　2.　専門職 (研究職，技師，コンピューターエンジニア，医師，看護師，教員など)  　3.　技術者 (電気技術者，コンピューター技術者，栄養士など)  　4.　事務職 (一般事務員，経理，秘書，パンチャーなど)  　5.　サービス (販売員，保安員，ウェイトレス，保育，介護者など)  　6.　技術を必要とする生産技能職 (建築，機械修理，整備，手工芸など)  　7.　機械を操作する生産技能職 (機械の運転・操作，自動車の運転など)  　8.　身体を使う作業の多い生産技能職 (包装，出荷，清掃など)  　9.　その他の仕事 | | | |
| 6．あなたの平均的な一週間の労働時間は何時間ですか。 （　　　　　）時間 | | | |
| 7. あなたの世帯収入（税込み）はおおよそいくらですか。 | | | 1．299万円以下  2．300-499万円  3．500-799万円  4．800-999万円  5．1000-1499万円  6．1500万円以上 |
| 8. あなたは子どもがいますか。 | | | 1. はい  2. いいえ |
| 9. あなたは住んでいる地域はどこですか。 | | | 1. 北海道  2. 東北  3. 関東  4. 中部  5. 近畿  6. 中国  7. 四国  8. 九州/沖縄 |
| 10. あなたの雇用形態は次のどれにあたりますか。 | | | 1. 経営者・役員  2. 正規の職員・従業員  3. パート/アルバイト  4. 契約社員  5. 労働者派遣事業所の派遣社員  6. 嘱託  7. その他 |
| 11. あなたは、障害者として特別な雇用枠で採用をされていますか。 | | 1. はい  2. いいえ | |
| 12. あなたが勤務している企業において常用労働者は何人ですか。あなたが勤務している事業所の他、本社、支社、工場、営業所など全ての国内の事業所を合わせた人数をお答え下さい。 | | 1. 10-29人  2. 30-49人  3. 50-99人  4. 100-299人  5. 300-999人  6. 1,000-4,999人  7. 5,000人以上  8. 官公庁 | |
| 13. あなたが勤務している事業所の事業内容を教えてください。 | | 1．農業，林業  2．漁業  3．鉱業，採石業，砂利採取業  4．建設業  5．製造業  6．電気・ガス・熱供給・水道業  7．情報通信業  8．運輸業，郵便業  9．卸売業，小売業  10．金融業，保険業  11．不動産業，物品賃貸業  12．学術研究，専門・技術サービス業  13. 宿泊業，飲食サービス業  14. 生活関連サービス業，娯楽業  15. 教育，学習支援業  16. 医療，福祉  17. 複合サービス事業  18. サービス業（他に分類されないもの）  19. 公務（他に分類されるものを除く）  20. 分類不能の産業 | |
